# Prediction of Phenolic Contents Based on Ultraviolet-B Radiation in Three-Dimensional Structure of Kale Leaves

**DOI:** 10.3389/fpls.2022.918170

**Published:** 2022-06-09

**Authors:** Hyo In Yoon, Jaewoo Kim, Myung-Min Oh, Jung Eek Son

**Affiliations:** ^1^Department of Agriculture, Forestry and Bioresources, Seoul National University, Seoul, South Korea; ^2^Division of Animal, Horticultural and Food Sciences, Chungbuk National University, Cheongju, South Korea; ^3^Research Institute of Agriculture and Life Sciences, Seoul National University, Seoul, South Korea

**Keywords:** antioxidants, flavonoids, growth stage, light interception, plant factory

## Abstract

Ultraviolet-B (UV-B, 280–315 nm) radiation has been known as an elicitor to enhance bioactive compound contents in plants. However, unpredictable yield is an obstacle to the application of UV-B radiation to controlled environments such as plant factories. A typical three-dimensional (3D) plant structure causes uneven UV-B exposure with leaf position and age-dependent sensitivity to UV-B radiation. The purpose of this study was to develop a model for predicting phenolic accumulation in kale (*Brassica oleracea* L. var. *acephala*) according to UV-B radiation interception and growth stage. The plants grown under a plant factory module were exposed to UV-B radiation from UV-B light-emitting diodes with a peak at 310 nm for 6 or 12 h at 23, 30, and 38 days after transplanting. The spatial distribution of UV-B radiation interception in the plants was quantified using ray-tracing simulation with a 3D-scanned plant model. Total phenolic content (TPC), total flavonoid content (TFC), total anthocyanin content (TAC), UV-B absorbing pigment content (UAPC), and the antioxidant capacity were significantly higher in UV-B-exposed leaves. Daily UV-B energy absorbed by leaves and developmental age was used to develop stepwise multiple linear regression models for the TPC, TFC, TAC, and UAPC at each growth stage. The newly developed models accurately predicted the TPC, TFC, TAC, and UAPC in individual leaves with *R*^2^ > 0.78 and normalized root mean squared errors of approximately 30% in test data, across the three growth stages. The UV-B energy yields for TPC, TFC, and TAC were the highest in the intermediate leaves, while those for UAPC were the highest in young leaves at the last stage. To the best of our knowledge, this study proposed the first statistical models for estimating UV-B-induced phenolic contents in plant structure. These results provided the fundamental data and models required for the optimization process. This approach can save the experimental time and cost required to optimize the control of UV-B radiation.

## Introduction

*Brassica* species are vegetables containing high levels of nutrients and health-promoting phytochemicals (Francisco et al., 2017). Kale (*B. oleracea* var. *acephala*) is a functional food that benefits human health, supported by much scientific evidence, and is a rich source of bioactive compounds such as polyphenols and carotenoids with antioxidant capacity (AOC; Šamec et al., 2019). The biosynthesis of such bioactive compounds is improved through biotic and abiotic elicitation (Thakur et al., 2019). Ultraviolet (UV) radiation, especially Ultraviolet-B (UV-B; 280–315 nm), has been reported as an effective elicitor (Loconsole and Santamaria, 2021). Contrary to the long-held opinion that UV-B radiation is predominantly harmful to plants, recent studies have highlighted that a low fluence rate of UV-B radiation triggers distinct changes in the secondary metabolism, resulting in bioactive compound accumulation such as phenolics and flavonoids (Schreiner et al., 2012). The accumulation of phenolics in epidermal tissues acts as a sunscreen, and their innate antioxidant potential protects underlying sensitive tissues from UV-B-induced damage (Takshak and Agrawal, 2019). However, the localized accumulation may complicate the distribution of phenolics depending on developmental age and uneven UV-B exposure with leaf position (Csepregi et al., 2017; Yoon et al., 2021a).

Ultraviolet-B-induced metabolic changes are multifaceted in terms of optical, morphological, and physiological factors; therefore, the use of UV-B radiation requires more precise manipulation to enhance the phenolic content (Schreiner et al., 2012). The actual dose perceived by the plant tissue depends not only on UV-B intensity from the light source but also on various optical factors and the morphological structure of the plant (Schreiner et al., 2012). Even under natural light, the attenuation of UV radiation in the plant canopy is affected by the spatial distribution and angle of the leaves, which are difficult to mathematically describe (Aphalo et al., 2012). Even without plants, lighting conditions with various optical factors, such as lighting distance, physical light distribution, and spectral power distribution of light sources, affect the spatial light distribution simulated in a growth chamber (Hitz et al., 2019). In addition, the simulated light absorbed by leaves depends on planting density and lighting arrangement as well as the three-dimensional (3D) structure of the plant in a controlled environment (Kim et al., 2020).

The developmental stage of plants is a strong determinant of the stress response and susceptibility to various environmental stresses (Rankenberg et al., 2021). In some cases, physiologically young leaves showed higher sensitivity to UV-B radiation, resulting in higher amounts of phenolics, higher AOC, or a higher expression of phenylpropanoid pathway genes (Majer and Hideg, 2012; Rizi et al., 2021). Furthermore, phenolic changes were more affected by the developmental stage than by UV-B levels in pak choi plants (Heinze et al., 2018). However, young leaves, typically located near the top of the plant, are exposed to targeted lighting conditions, while older leaves rely on light penetration in the 3D structure of plants. The effect of the developmental stage cannot be separated from the positional light-exposure effect. For experimental purposes, positional UV-exposure effects can be avoided by selecting plants with a 2D structure (Majer and Hideg, 2012; Csepregi et al., 2017). The UV dose absorbed by the leaves in the 3D structure has been attempted to be quantified through simulation with a 3D-scanned plant model, and the UV exposure and developmental effects could be separately analyzed (Yoon et al., 2021a,b).

For the application of UV-B radiation to phenolic production, a common strategy is to find a combination of UV-B-related factors, such as UV-B dose (fluence rate and duration), timing (plant developmental stage at which UV-B exposure is initiated), or wavelength/type of UV-B (Schreiner et al., 2009; Sun et al., 2010; Rechner et al., 2016; Dou et al., 2019). Traditional UV-B lamps are prone to causing photosynthetic damage to plants due to their broadband wavelength range, including shorter wavelengths close to UV-C (100–280 nm) and excessive and difficult-to-control energy (Mosadegh et al., 2019; Yoon et al., 2020). Recently, the performance of light-emitting diodes (LEDs) has advanced enough to provide light of the desired wavelength and intensity for plants (Kneissl et al., 2019; Loi et al., 2020; Paradiso and Proietti, 2021). Narrowband UV-B LEDs have also been applied at low doses to enhance health-promoting compound accumulation without damaging plants (Wiesner-Reinhold et al., 2021). Since the application of UV-B radiation incurs additional energy costs, either UV-B energy efficiency or maximum phenolic production should be pursued in controlled environmental agriculture. Prediction of UV-B-induced phenolic content will allow us to find the optimal UV-B conditions for maximizing phenolic production.

Statistical modeling has widely been used because it is simple but powerful for predicting and quantifying the relationship between variables (Kim et al., 2016). The plant developmental stage affects plant structures as well as their sensitivity to UV-B radiation (Yoon et al., 2021b). Thus, the phenolic production could be quantified with the modeling based on developmental age and UV-B radiation on the plant structure. This study focused on preharvest UV-B exposure as an elicitor for the biosynthesis of phenolics in non-acclimated plants to rule out possible UV-B acclimation effects (Liao et al., 2020). This study aimed to analyze UV-B-induced phenolic accumulation with preharvest UV-B exposure based on UV-B radiation interception and developmental stage of plants, and ultimately aimed to develop statistical models and investigate the UV-B energy yield for the phenolic accumulation. For this purpose, the contents of phenolics, flavonoids, anthocyanin, UV-absorbing pigments, and chlorophylls were evaluated in individual leaves with UV-B radiation interception simulated with 3D-scanned plant models according to growth stage.

## Materials and Methods

### Plant Materials and Experimental Conditions

Kale (*B. oleracea* L. var. *acephala*) seeds were sown on sponge cubes in water culture under fluorescent lamps at a photosynthetic photon flux density (PPFD) of 150 μmol m^–2^ s^–1^ over the waveband 400–700 nm for a 16-h light period (Yoon et al., 2021a,b). After true leaves appeared, seedlings were supplied with a nutrient solution for *Brassica* modified from a previous study (Choi et al., 2005): N 137.8, P 30.9, K 140.9, Ca 104.6, Mg 54.8, Fe 2.76, Cu 0.02, Zn 0.05, Mn 0.68, B 0.50, and Mo 0.01 mg L^–1^, at an electrical conductivity (EC) of 0.6 dS m^–1^. After the fourth true leaf appeared, the seedlings of uniform size were transplanted into plant factory modules with a deep flow technique system. The modules were maintained at an air temperature of 19–22°C, relative humidity of 65–75%, a CO_2_ concentration of 500 μmol mol^–1^, an EC of 1.18–1.22 dS m^–1^, and a pH of 6.8–7.0. The plants were irradiated with red, blue, and white LEDs at a PPFD of 255 μmol m^–2^ s^–1^ (at 7 cm from the center of the ground) for a 16-h light period. In kale plants grown in plant factories (Yoon et al., 2019) or fields (Hagen et al., 2009) under normal conditions, the concentration of bioactive compounds was not significantly different depending on the harvest time. Therefore, the harvest time was determined as 4 weeks when the total fresh mass reached around 100 *g*, which is sufficient for sale (Supplementary Figure 1). The four plants per treatment were harvested separately at 23, 30, and 38 days after transplanting (DAT).

**FIGURE 1:**
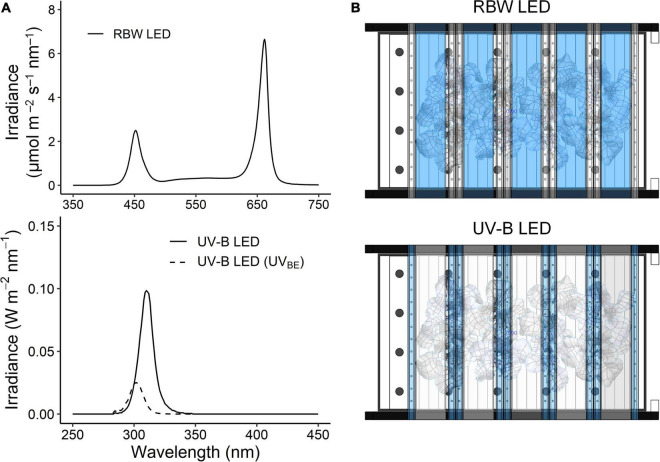
Spectra **(A)** and arrangement **(B)** of red, blue, and white light-emitting diodes (RBW LED) and UV-B LED used in the plant factory. The irradiance of UV-B LEDs with a spectral peak at about 310 nm was 3.0 W m^– 2^ at 280–400 nm. UV-B_*BE*_ indicates biologically effective UV-B calculated using a biological spectral weighting function for plants (Flint and Caldwell, 2003), and thereby its spectral peak was at about 302 nm. The RBW LEDs irradiated at a photosynthetic photon flux density of 255 μmol m^– 2^ s^– 1^.

For UV treatment, UV-B LEDs with a spectral peak at approximately 310 nm were used, and the irradiance at 7 cm above the center of the bottom was 3.0 W m^–2^. The spectrum and intensity of photosynthetically active radiation (PAR) and UV-B LEDs were measured using a spectroradiometer (Blue-Wave spectrometer, StellarNet Inc., Tampa, FL, United States) in the range of 250–900 nm (Figure 1A). The plants at 23, 30, and 38 DAT were irradiated with UV-B LEDs for 6 or 12 h (UV6h or UV12h) and then harvested after recovery for 4 h. The UV6h and UV12h treatments corresponded to 15.6 and 31.3 kJ m^–2^ day^–1^ biologically effective UV-B radiation (UV-B_*BE*_), respectively, calculated using a biological spectral weighting function for plants (Flint and Caldwell, 2003). The arrangements of PAR and UV-B LEDs are shown in Figure 1B.

### Three-Dimensional-Scanning to Optical Simulation for Radiation Interception Analysis

**FIGURE 2:**
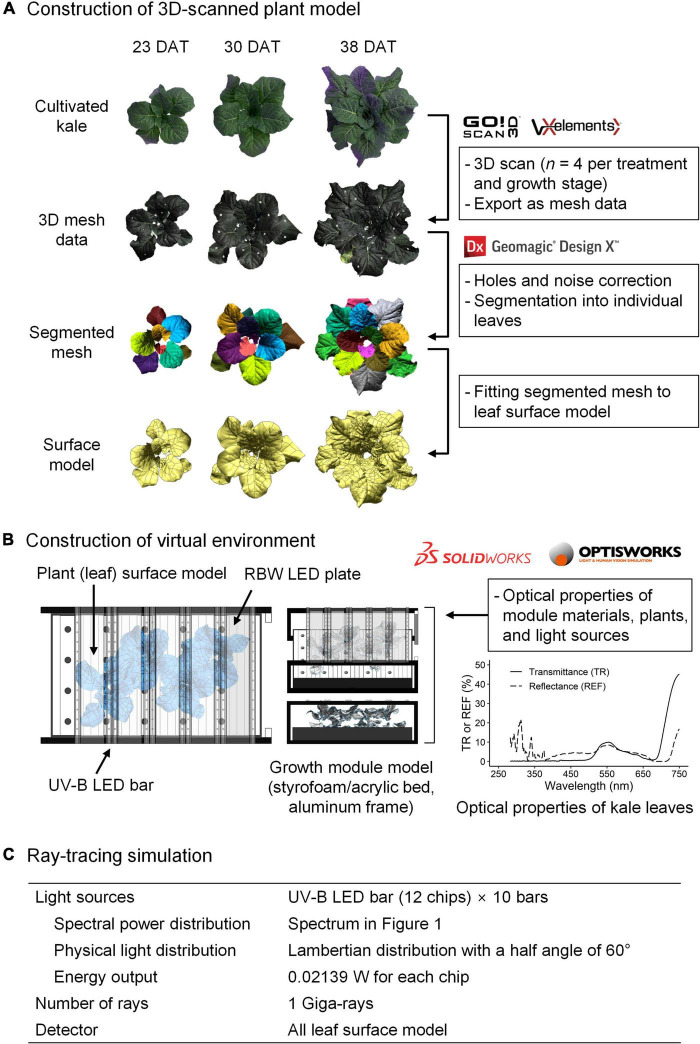
Schematic diagram of 3D scanning to optical simulation for radiation interception analyzed in kale plants. After the construction of a 3D-scanned plant model **(A)**, the virtual environment for simulation **(B)** consisted of a 3D model of the growth module, light sources, and plants with actual dimensions and arrangement. A ray-tracing simulation **(C)** was conducted with these parameters. TR, transmittance; REF, reflectance.

Ultraviolet radiation interception analysis with a 3D-scanned plant model and ray-tracing simulation was performed as previously described (Kim et al., 2020; Yoon et al., 2021a; Figure 2). In brief, plant models were directly obtained using a high-resolution portable 3D scanner (Go! SCAN50TM, Creaform Inc., Lévis, QC, Canada) and its software (Vxelement, Creaform Inc.). Four plants per treatment were 3D-scanned after the UV treatment at 23, 30, and 38 DAT. After repair for holes and noise, the 3D mesh data were segmented into leaf mesh data and reconstructed into individual surface models using reverse engineering software (Geomagic Design X, 3D Systems, Rock Hill, SC, United States). The virtual plant factory modules based on the dimensions measured were constructed using 3D computer-aided design software (Solidworks, Dassault Systèmes, Vélizy-Villacoublay, France). The 3D model arrangement and simulation parameters, including the optical properties of materials and plants, and the setting of light sources and detectors, are described in Figure 2. All 3D models were placed in the same position and orientation as the actual materials and plants. The ray-tracing simulation was performed using ray-tracing software (Optisworks, Optis Inc., La Farlède, France). The daily UV-B radiation interception on individual leaves was calculated from the simulation results with UV-exposure durations.

### Growth Characteristics

Growth parameters, including total fresh mass, total leaf area, and root dry mass, were measured as shown in Supplementary Figure 1. Fresh masses of individual leaves were measured separately at harvest with four plants per treatment (Supplementary Figure 2). After photographing all leaves, the areas of individual leaves were calculated with an image analysis software (ImageJ 1.53, National Institutes of Health, Bethesda, MD, United States). Leaf order was determined as an absolute order of emergence (the first true leaf was set as 1) and was numbered from the oldest leaf at the bottom to the youngest at the top of the plant. Leaf groups at each growth stage were determined based on the relative growth rate of individual leaves, which are shown in Supplementary Figure 3 and Supplementary Table 1 (Behn et al., 2011; Pontarin et al., 2020). Leaf groups 1, 2, and 3 corresponded to leaf orders 1–3, 4–7, and 8–11, respectively, at 23 DAT, leaf orders 3–6, 7–9, and 10–14, respectively, at 30 DAT, and leaf orders 4–8, 9–12, and 13–18, respectively, at 38 DAT.

### Phenolic Content, Antioxidant Capacity, and Photosynthetic Pigment Content

#### Sample Preparation

The whole leaves were sampled separately at harvest and frozen at –80°C. After lyophilization with a freeze dryer (FD8512, Ilshin Biobase Co., Yangju, South Korea) at –80°C under a vacuum of 0.007 mm Hg for 120 h, the leaf dry mass was determined. The lyophilized sample was pulverized in liquid nitrogen using a cryogenic grinder (SPEX 6875D Freezer/Mill, SPEX SamplePrep, Metuchen, NJ, United States), and then stored in the dark at 4°C until needed for analysis. The freeze-milled samples used for the analysis corresponded to leaf orders 5–9, 5–13, and 5–17 at 23, 30, and 38 DAT, respectively.

#### Total Phenolic Content and Total Flavonoid Content

For determining Total phenolic content (TPC) and total flavonoid content (TFC), aliquots of the powdered sample (50 mg) were mixed with 1 ml of 80% (v/v) methanol, incubated in the dark at 4°C for 18 h, and then centrifuged at 11,000 × *g* at 4°C for 10 min. TPC was determined according to the Folin–Ciocalteu colorimetric method (Ainsworth and Gillespie, 2007). The supernatant of 100 μl was collected in a 2-ml tube and mixed with 200 μl of 10% (v/v) Folin–Ciocalteu solution (Junsei Chemical Co., Ltd., Tokyo, Japan), and 400 μl of distilled water. After vortex mixing, 800 μl of 700 mM Na_2_CO_3_ was added. The mixture was shaken for 10 s and incubated in a water bath at 45°C for 15 min. The absorbance was read at 765 nm using a spectrophotometer (Photolavis, WTW, Weilheim, Germany), and the TPC was expressed as milligrams of gallic acid (Sigma–Aldrich Chemical Corp., St. Louis, MO, United States) equivalent per gram of dry mass (mg GAE g^–1^ DM). TFC was determined according to the aluminum chloride colorimetric method (Dewanto et al., 2002). The supernatant of 100 μl was collected in a 2-ml tube, and mixed with 500 μl of distilled water and 30 μl of 5% (w/v) NaNO_2_. After 6 min, 60 μl of 10% (w/v) AlCl_3_ was added. After 5 min, 200 μl of 1 M NaOH and 110 μl of distilled water were added, and all reactants were thoroughly mixed. After incubating for 5 min, the absorbance was read at 510 nm, and the TFC was expressed as milligrams of catechin acid (Supelco, Bellefonte, PA, United States) equivalent per gram of dry mass (mg CE g^–1^ DM).

#### Total Anthocyanin Content and UV-Absorbing Pigment Content

For determining total anthocyanin content (TAC) and UV-B absorbing pigment content (UAPC), the aliquots of the powdered sample (20 mg) were mixed with 1 ml of 1% acidified methanol, incubated in the dark at 4°C for 48 h, and then centrifuged at 11,000 × *g* at 4°C for 15 min. The supernatant of the extract was diluted two-fold, and the absorbance was read at 530 and 657 nm (A_530_ and A_657_, respectively). TAC was determined with the corrected absorbance as A_530_, 0.25A_657_ (Sytar et al., 2018) and expressed as milligrams of cyanidin 3-glucoside (Sigma–Aldrich Chemical Corp.) equivalent per gram of dry mass (mg C3GE g^–1^ DM). The supernatant of the extract was diluted 20-fold, and the absorbance was read at 285 and 330 nm (Si et al., 2015; Khudyakova et al., 2019). UAPC was determined as the average of two absorbances and expressed as an absorbance per gram of dry mass (OD g^–1^ DM).

#### Antioxidant Capacity

Antioxidant capacity can be determined depending on the choice of the assay, and analyzing phenol-rich samples by a single assay is recommended to be avoided (Csepregi et al., 2016). Thus, both the 2, 2’-azino-bis (3-ethylbenzothiazoline-6-sulfonic acid)-diammonium salt (ABTS) assay and the 2, 2-diphenyl-1-picrylhydrazyl (DPPH) assay were performed. The powdered sample of 50 mg was mixed with 1 ml of 80% (v/v) methanol, and the mixture was incubated in the dark at 4°C for 42 h and then centrifuged at 11,000 × *g* at 4°C for 10 min. The ABTS radical scavenging activity was determined as described by Re et al. (1999). Briefly, the ABTS radical cation (ABTS^+^) reagent was produced by reacting 7 mM ABTS solution (Sigma–Aldrich Chemical Corp.) with 2.45 mM K_2_S_2_O_8_ (1:1, v/v), and stored in the dark at 4°C for 18 h before use. The ABTS^+^ solution was diluted with 80% methanol to obtain appropriate absorbance. The supernatant of 50 μl was added to 1.8 ml of diluted ABTS^+^ solution. After 6 min of incubation in the dark at room temperature, the absorbance was read at 734 nm. The DPPH radical scavenging activity was determined as described by Brand-Williams et al. (1995). DPPH was purchased from Alfa Aesar (Ward Hill, MA, United States), and the DPPH solution was freshly made in methanol before use. The supernatant of 100 μl was added to 1.8 ml of 0.12 mM DPPH methanol solution. After incubation in the dark at room temperature for 30 min, the absorbance was read at 517 nm. A calibration curve for ABTS and DPPH assays was constructed using L-ascorbic acid (Samchun Pure Chemical Co., Ltd., Pyeongtaek, South Korea). The AOC_*ABTS*_ and AOC_*DPPH*_ were expressed as milligrams of ascorbic acid equivalent per gram of dry mass (mg AAE g^–1^ DM).

#### Photosynthetic Pigment Content

For determining photosynthetic pigment, the aliquots of the powdered sample (50 mg) were mixed with 1 ml of 80% (v/v) acetone, incubated in the dark at room temperature for 24 h, and then centrifuged at 11,000 × *g* for 10 min. Chlorophyll *a*, Chlorophyll *b*, and carotenoid contents were calculated based on absorbance at 663, 647, and 470 nm according to the method of Lichtenthaler and Buschmann (2001) and expressed as milligrams of chlorophyll or carotenoid per gram of dry mass (mg g^–1^ DM).

### Multiple Regression Model and Ultraviolet-B Yield

Total phenolic content, TFC, TAC, UAPC, and AOC per leaf were calculated by multiplying their concentrations (mg eq. g^–1^ DM) by the leaf dry mass (g DM). Stepwise multiple linear regressions were used to develop statistical models for predicting phenolic accumulation. The model was obtained by stepwise regression using the backward elimination method based on a second-order multi-polynomial (quadratic) model, including the single effect of leaf order and UV radiation interception and the interaction effect:


(1) $$\text{M}\,\left(L,U\right)=\beta_{0}+\beta_{1}\,\text{L}+\beta_{2}\,{\text{L}}^{2}+\beta_{3}\,\text{U}+\left(\beta_{4}\,\text{L}+\beta_{5}\,{\text{L}}^{2}\right)\,\text{U}$$

where *M* is the phenolic content per leaf (mg eq. per leaf), *L* is leaf order numbered as an absolute order of leaf emergence, *U* is the daily UV radiation interception per leaf (kJ day^–1^ per leaf), and β_0_–β_5_ are the regression coefficients obtained by the regression analysis at each growth stage. The UV-B energy yield for phenolic accumulation per leaf was determined as the change in the phenolic content per absorbed UV-B energy and calculated as the slope of the multiple regression surface against UV interception as follows:


(2) $$\text{Y}\,\left(L,U\right)=\left[\text{M}\,\left(L,U\right)-\text{M}\,\left(L,0\right)\right]/\text{U}=\beta_{3}+\beta_{4}\,\text{L}+\beta_{5}$$

where *Y* is UV-B energy yield for phenolic accumulation per leaf (mg eq. kJ^–1^ day) at each *L* and *U*.

### Statistical Analysis

For all data, the homogeneity of variance was evaluated using Levene’s test, and the normality was evaluated using the Shapiro–Wilk test. The mean values were compared using one-way or two-way ANOVA and Tukey’s honestly significant difference (HSD) test to assess the effects of the UV-B treatment (control, UV6h, and UV12h), leaf group 1–3, or growth stage (23, 30, and 28 DAT). For data that failed the normality test, the Kruskal–Wallis and Dunn’s tests were used, and for data that failed the homogeneity of variance, the Welch’s ANOVA and Games-Howell tests were used.

For model development, nine plants per growth stage were used for the data set, which corresponded to a total of 250 data points, including 66, 74, and 110 data points at 23, 30, and 38 DAT, respectively, after removing outliers with the interquartile range. For accuracy of the multiple regression model, a data set (not used to develop the model) including nine plants and 76 data points was used for validation. The coefficient of determination (*R*^2^), root mean squared error (RMSE), and normalized RMSE were selected. All visualization and statistical analyses were performed using R software (R 4.0.2, R Foundation, Vienna, Austria).

## Results

### Ultraviolet-B Radiation Interception According to Leaf Group and Growth Stage

**FIGURE 3:**
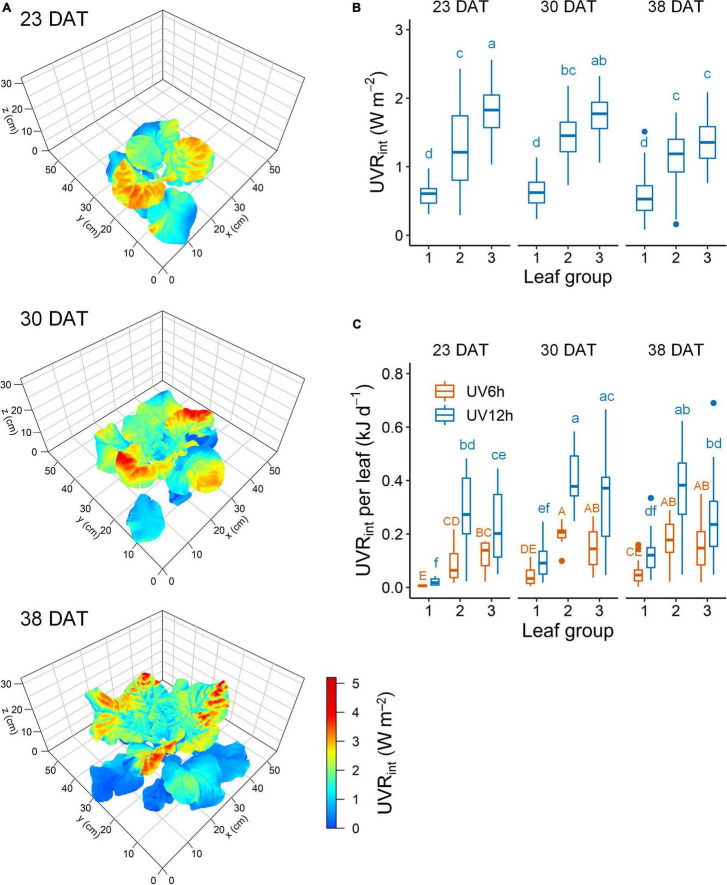
Representative simulated UV-B radiation interception (UVR_*int*_) on 3D-scanned models **(A)**, mean UVR_*int*_ **(B)**, and daily UVR_*int*_ per leaf **(C)** of kale plants according to UV-B radiation and leaf group at 23, 30, and 38 days after transplanting (DAT). Leaf groups were determined according to the order of leaf emergence and their relative growth state (Supplementary Figure 3 and Supplementary Table 1). Different letters indicate significant differences at *P* < 0.05 for UVR_*int*_ value (*n* = 26–47) and daily UVR_*int*_ per leaf (*n* = 11–25) with growth stage and leaf group by two-way ANOVA and Tukey’s HSD test.

Ultraviolet-B radiation interception with a 3D-scanned plant model was simulated along with the actual plant structure at each growth stage (Figure 3A). The UV radiation interception (UVR_*int*_) was significantly higher in the order of leaf groups 3, 2, and 1 (Figure 3B). Across all stages, the UVR_*int*_ was significantly lower by 51.7% in leaf group 1 and significantly higher by 31.4% in group 3 than in group 2. At 23, 30, and 38 DAT, the mean UVR_*int*_ was 1.20, 1.28, and 1.05 W m^–2^, respectively. The daily UVR_*int*_ per leaf did not significantly differ between the leaf groups 2 and 3 and was affected by individual leaf area (Figure 3C). Across all data, the daily UVR_*int*_ per plant was significantly higher with growth stage (1.7 ± 0.5, 2.2 ± 0.3, and 2.6 ± 0.4 kJ day^–1^ per plant at 23, 30, and 38 DAT, respectively).

### Age-Dependent Changes in Phenolic Content According to Ultraviolet-B Radiation

**FIGURE 4:**
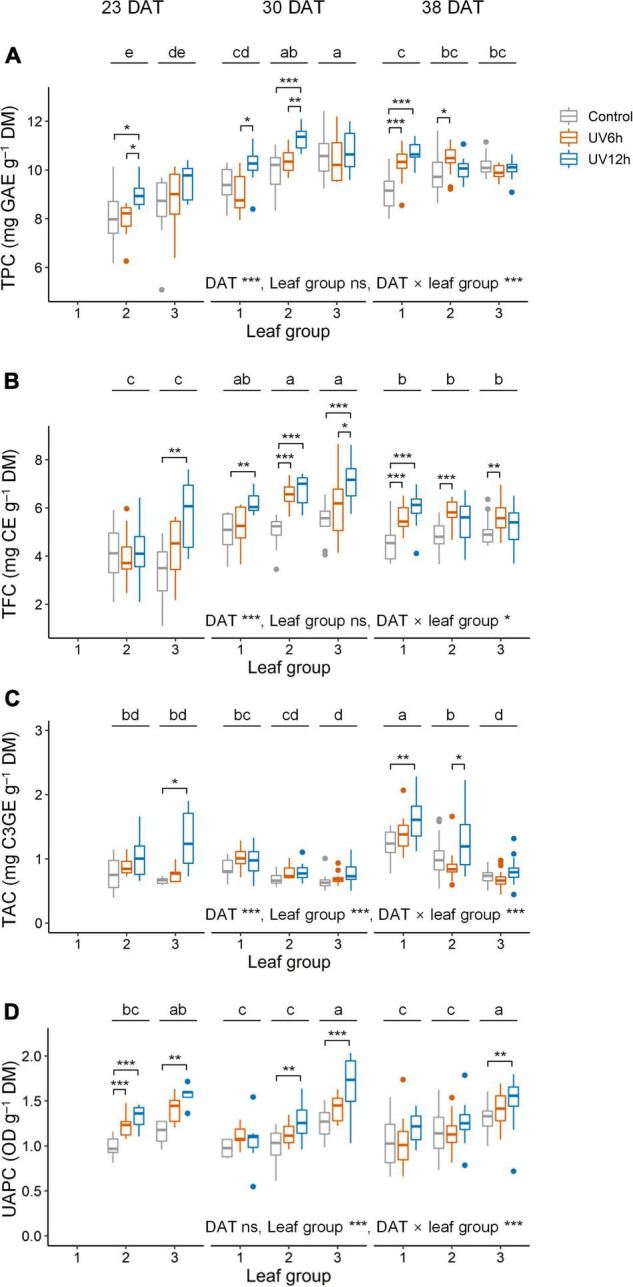
Total phenolic content (TPC, **A)**, total flavonoid content (TFC, **B)**, total anthocyanin content (TAC, **C)**, and UV-absorbing pigment content (UAPC, **D)** in individual leaves of kale plants according to UV-B radiation and leaf group at 23, 30, and 38 days after transplanting (DAT). Leaf groups were determined according to the order of leaf emergence and their relative growth state (Supplementary Figure 3 and Supplementary Table 1). Asterisks indicate significant differences between UV-B treatments at each growth stage and leaf group by one-way ANOVA and *post-hoc* test, *P < 0.05; **P < 0.01; ***P < 0.001; *n* = 7–22. Different letters indicate significant differences among growth stage and leaf group at *P* < 0.05 by two-way ANOVA and *post-hoc* test (*n* = 22–59) referring to the “Materials and Methods” section.

Total phenolic content, TFC, TAC, and UAPC were significantly affected by UV-B radiation, growth stage, and leaf groups (Figure 4). Across all data, the contents of all the compounds were significantly higher in the UV12h treatment than in the control. TPC and TFC were significantly higher in the order of 30, 38, and 23 DAT. They were the highest in leaf groups 2 and 3 at 30 DAT, respectively, in UV12h treatment (13.2 and 31.6% higher, respectively, than in the control).

**FIGURE 5:**
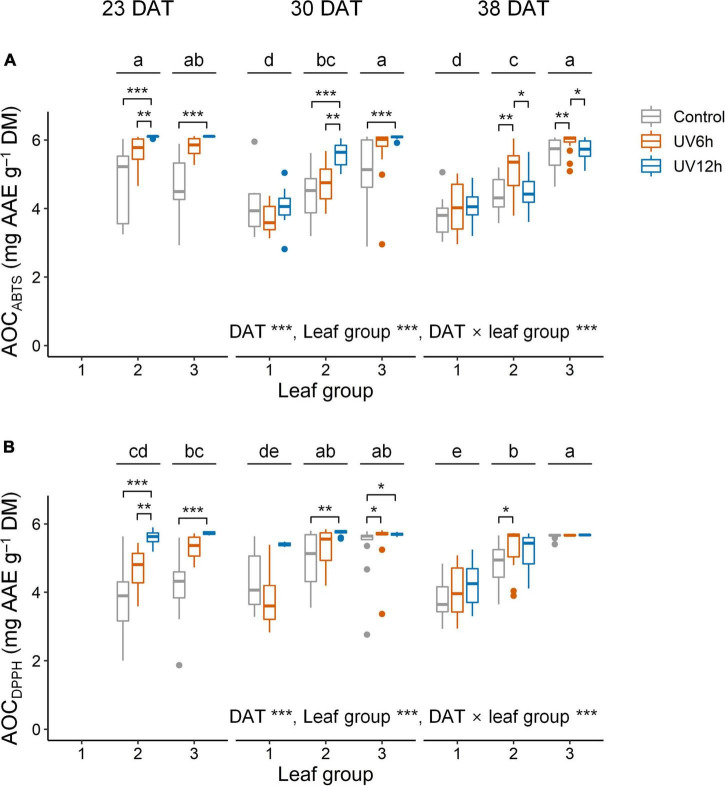
Antioxidant capacity using ABTS (AOC_*ABTS*_, **A)** and DPPH assays (AOC_*DPPH*_, **B)** in individual leaves of kale plants according to UV-B radiation and leaf group at 23, 30, and 38 days after transplanting (DAT). Leaf groups were determined according to the order of leaf emergence and their relative growth state (Supplementary Figure 3 and Supplementary Table 1). Asterisks indicate significant differences between UV-B treatments at each growth stage and leaf group by one-way ANOVA and *post-hoc* test, *P < 0.05; **P < 0.01; ***P < 0.001; *n* = 7–22. Different letters indicate significant differences among growth stage and leaf group at *P* < 0.05 by two-way ANOVA and *post-hoc* test (*n* = 22–59) referring to the “Materials and Methods” section.

Antioxidant capacity using ABTS and the AOC_*DPPH*_ were significantly higher in the UV12 treatment and leaf group 3 than in the others (Figure 5). The AOC_*ABTS*_ and AOC_*DPPH*_ were positively correlated with the UAPC, and their Pearson’s correlation coefficients were 0.69 and 0.62, respectively, at *P* < 0.001 (data not shown). In contrast, the UAPC, AOC_*ABTS*_, and AOC_*DPPH*_ were significantly higher in the order of leaf groups 3, 2, and 1, and the TAC was significantly higher in the reverse order. The UAPC was the highest in leaf group 3 at 30 DAT in the UV12h treatment (34.2% higher than in the control).

**FIGURE 6:**
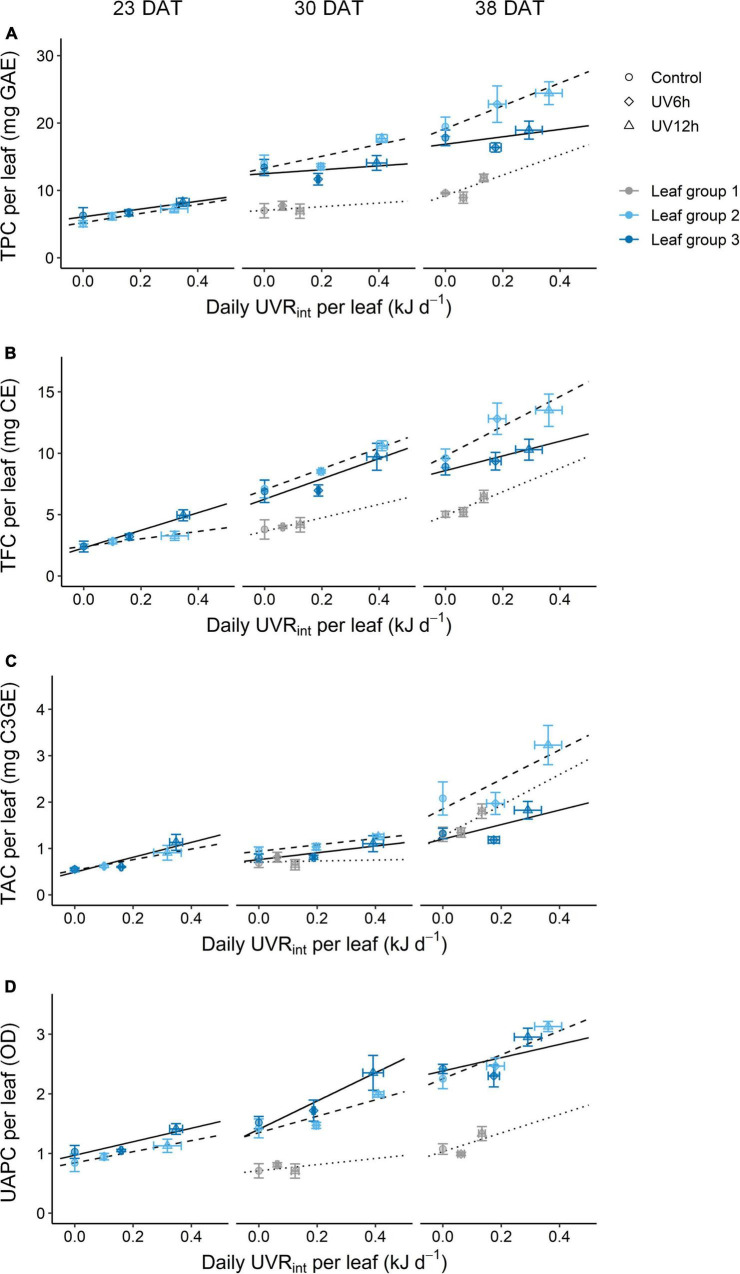
Relationships between phenolic content and the daily UV-B radiation interception (UVR_*int*_) per leaf of kales according to leaf group at 23, 30, and 38 days after transplanting (DAT); total phenolic content (TPC, **A)**, total flavonoid content (TFC, **B)**, total anthocyanin content (TAC, **C)**, and UV-absorbing pigment content (UAPC, **D)** in individual leaves. Leaf groups were determined according to the order of leaf emergence and their relative growth state (Supplementary Figure 3 and Supplementary Table 1). The vertical and horizontal bars indicate SE (*n* = 4). The lines show linear fits of the leaf group 3, 2, and 1 data sets (solid, dashed, and dotted lines).

The TPC, TFC, TAC, and UAPC in individual leaves were increased with daily UVR_*int*_ per leaf, and the slopes were dependent on growth stage and leaf group (Figure 6). The slopes, i.e., the increase rates of TPC and TFC with daily UVR_*int*_ per leaf in individual leaves, were highest in leaf group 2 at 38 DAT, followed by 30 DAT. Those of TPC and TFC in individual groups were increased as growth progressed. In contrast, the increase rates of TAC and UAPC were the highest in leaf group 3 at 23–30 DAT, and those were lower in leaf group 3 than in the others at 38 DAT.

### Prediction Models of Phenolic Content Based on Ultraviolet-B Radiation Interception and Leaf Order According to Growth Stage

**FIGURE 7:**
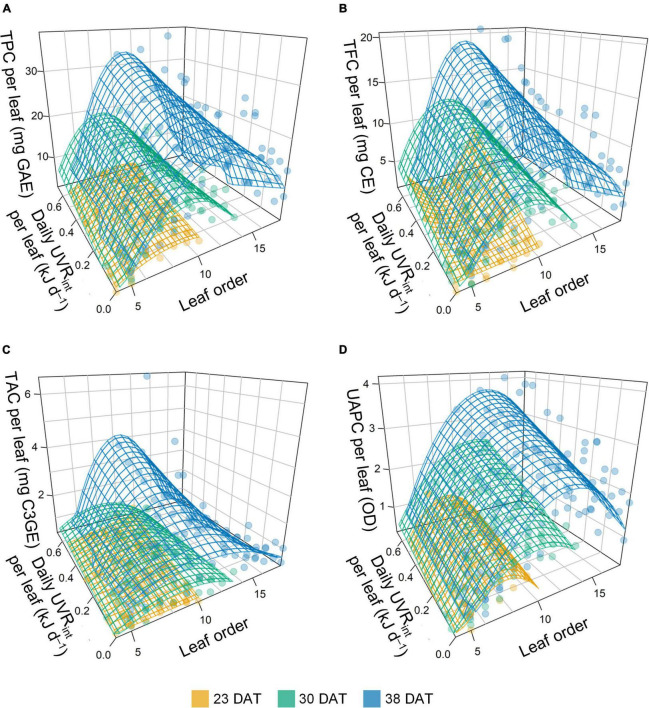
Stepwise multiple linear regression model for phenolic content per leaf of kale plants with daily UV-B radiation interception (UVR_*int*_) per leaf and leaf order at 23, 30, and 38 days after transplanting (DAT): TPC, total phenolic content **(A)**; TFC, total flavonoid content **(B)**; TAC, total anthocyanin content **(C)**; and UAPC, UV-absorbing pigment content **(D)**. The surfaces were obtained by stepwise multiple regression models referring to Eq. 1 and Table 1.

**FIGURE 8:**
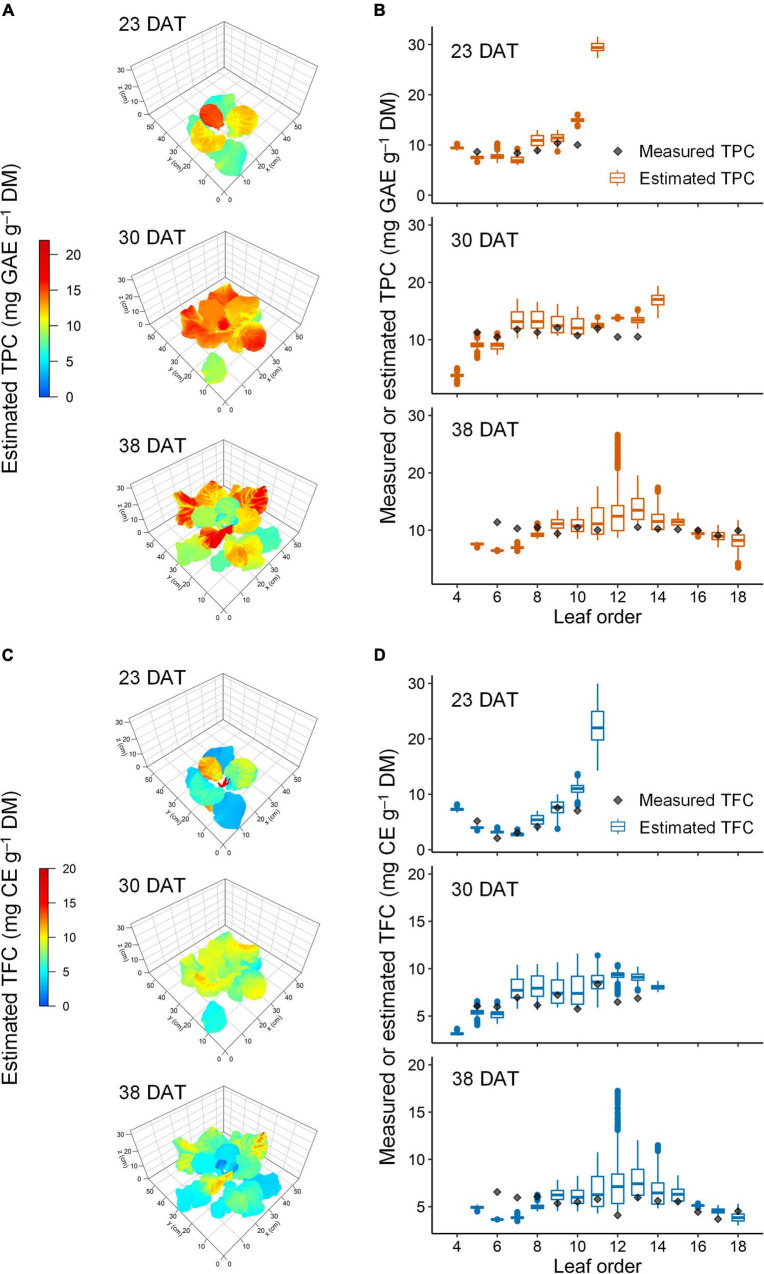
Representative estimated distribution of total phenolic content (TPC, **A,B)** and total flavonoid content (TFC, **C,D)** in the leaves of kale plants under 12-h UV-B exposure at 23, 30, and 38 days after transplanting (DAT). The multiple regression models refer to in Figure 4 and Table 1, and the 3D-scanned plant model refers to in Figure 3.

Multiple regression models for the phenolic contents per leaf were developed based on daily UVR_*int*_ per leaf and leaf order according to growth stage, and all models were significant at *P* < 0.001 (Figure 7 and Table 1). The models for the TPC, TFC, and UAPC in individual leaves at each growth stage showed a high explanatory power, with *R*^2^ = 0.62–0.79. However, the models for the TAC per leaf showed a low explanatory power, with *R*^2^ < 0.51. All estimated coefficients were significant for the regression models. From the models, the spatial and intraindividual distributions of TPC and TFC on the 3D-scanned plant model were estimated (Figure 8). The *R*^2^ values for the four models were higher in the data set integrated from the models across the whole growth stage than in the models at each growth stage (Table 1 and Supplementary Figure 5).

**TABLE 1:** Stepwise multiple linear regression results and models to predict phenolic content per leaf of kale plants at 23, 30, and 38 days after transplanting (DAT) based on leaf order (*L*) and daily UV-B radiation interception per leaf (*U*) referring to Eq. 1.

Model	DAT	*R* ^2^	Adj *R*^2^	RMSE	NRMSE (%)	*p-value*	Model equation
TPC	23	0.63	0.61	1.23	17.3	<0.001	*M(L, U)* = –5.01 + 2.72 *L* – 0.16*L*^2^ + 6.54*U*
	30	0.77	0.76	2.17	18.4	<0.001	*M(L, U)* = –25.29 + 8.30 *L* – 0.44*L*^2^ + (4.54 *L* – 0.38*L*^2^)*U*
	38	0.72	0.70	3.90	22.3	<0.001	*M(L, U)* = –26.03 + 7.62 *L* – 0.31*L*^2^ – 73.52 *U* + (17.18 *L* – 0.80*L*^2^)*U*
	Integration	0.83		2.91	22.2		
TFC	23	0.62	0.60	0.73	21.4	<0.001	*M(L, U)* = 2.42 + 39.67 *U* + (–12.02 *L* + 0.96*L*^2^)*U*
	30	0.78	0.77	1.35	19.6	<0.001	*M(L, U)* = – 3.38 + 4.52 *L* – 0.25*L*^2^ + (2.34 *L* – 0.15*L*^2^)*U*
	38	0.74	0.73	2.16	23.3	<0.001	*M(L, U)* = –12.23 + 3.80 *L* – 0.16*L*^2^ -47.53 *U* + (10.88 *L* – 0.49*L*^2^)*U*
	Integration	0.83		1.65	23.5		
TAC	23	0.39	0.38	0.25	34.4	<0.001	*M(L, U)* = 0.48 + 0.18*LU*
	30	0.46	0.43	0.24	26.0	<0.001	*M(L, U)* = –1.21 + 0.48 *L* – 0.03*L*^2^ + (0.30 *L* – 0.03*L*^2^)*U*
	38	0.51	0.48	0.65	36.9	<0.001	*M(L, U)* = –0.44 + 0.41 *L* – 0.02*L*^2^ –17.19 *U* + (3.92 *L* – 0.19*L*^2^)*U*
	Integration	0.66		0.47	37.9		
UAPC	23	0.63	0.59	0.20	17.9	<0.001	*M(L, U)* = –3.18 + 1.13 *L* – 0.08*L*^2^ + 1.05*U*
	30	0.79	0.78	0.25	17.7	<0.001	*M(L, U)* = –1.78 + 0.64 *L* – 0.03*L*^2^ + 0.17*LU*
	38	0.72	0.71	0.48	23.1	<0.001	*M(L, U)* = –3.67 + 0.99 *L* – 0.04*L*^2^ + 0.14*LU*
	Integration	0.79		0.38	22.6		

*TPC, total phenolic content per leaf (mg GAE per leaf); TFC, total flavonoid content per leaf (mg CE per leaf); TAC, total anthocyanin content per leaf (mg C3GE per leaf); and UAPC, UV-absorbing pigment content per leaf (OD per leaf).*

*R^2^, the coefficient of determination; Adj R^2^, adjusted R^2^; RMSE, root mean squared error; and NRMSE, normalized RMSE (%).*

*Integration indicates results in the data set across all growth stages integrated from the models at each growth stage.*

**FIGURE 9:**
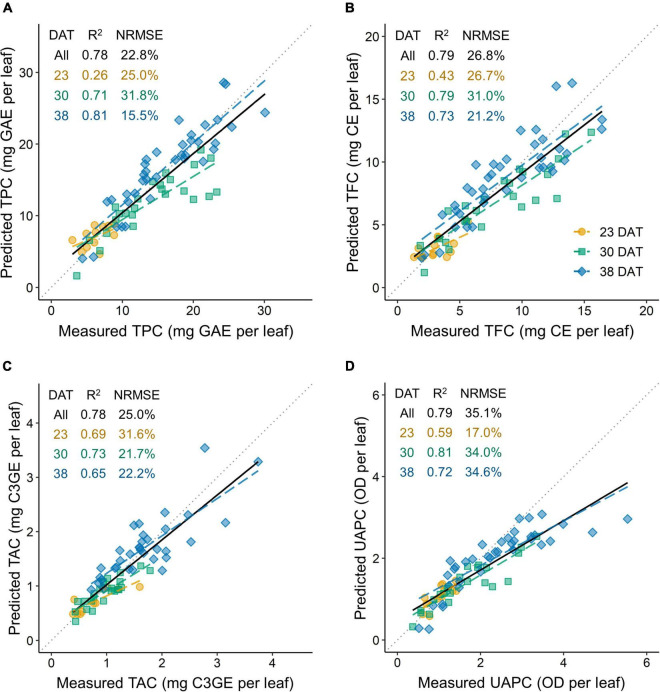
Model accuracy for predicting phenolic content per leaf of kale plants using a test data set: TPC, total phenolic content per leaf **(A)**; TFC, total flavonoid content per leaf **(B)**; TAC, total anthocyanin content per leaf **(C)**; and UAPC, UV-absorbing pigment content per leaf **(D)**. The coefficient of determination (*R*^2^) and the normalized root mean squared error (NRMSE) are presented inside each panel. The multiple regression models at 23, 30, and 38 days after transplanting (DAT) are shown in Figure 4 and Table 1. All in DAT indicate the results in the data set across all growth stages integrated from the models at each growth stage.

The accuracies of the models for TPC, TFC, TAC, and UAPC per leaf were validated using test data sets (Figure 9). All models performed well for the whole growth stage, with *R*^2^ = 0.78–0.79. The TPC, TFC, and UAPC models showed high performance with *R*^2^ > 0.7, except for those at 23 DAT. The TAC model showed high performance with *R*^2^ > 0.6 at all growth stages. The TPC model for the whole growth stage showed approximately 22.8% relative error, meaning that the model could predict the TPC per leaf with 77.2% accuracy during 23–38 DAT. Likewise, the models could predict TFC, TAC, and UAPC per leaf with 73.2, 75.0, and 64.9% accuracy, respectively, during 23–38 DAT.

### Ultraviolet-B Energy Yields for Phenolic Content With Growth Stage

The UV-B energy yields for the TPC, TFC, TAC, and UAPC based on absorbed UV energy were significantly different depending on the growth stage and leaf group (Table 2).

**TABLE 2:** UV-B energy yields for phenolic content per leaf of kale plants with leaf group at 23, 30, and 38 days after transplanting (DAT) referring to Eq. 2 and Table 1.

DAT	Leaf group	UV-B energy yield			
		
		TPC (mg GAE kJ^–1^ day)	TFC (mg CE kJ^–1^ day)	TAC (mg C3GE kJ^–1^ day)	UAPC (OD kJ^–1^ day)
23	2	6.54 c	3.14 d	1.05 bc	1.05 e
	3	6.54 c	7.68 b	1.53 b	1.05 e
30	1	13.1 b	7.91 b	0.89 bc	0.87 f
	2	11.6 b	8.70 b	0.81 bc	1.33 d
	3	2.47 c	6.63 bc	0.23 cd	1.86 b
38	1	3.80 c	2.16 d	0.32 cd	0.95 ef
	2	17.9 a	12.2 a	3.31 a	1.53 c
	3	3.65 c	5.05 cd	-0.28 d	2.15 a
DAT	23	6.54 B	4.91 B	1.22 A	1.05 C
	30	8.47 A	7.67 A	0.61 B	1.40 B
	38	8.02 A	6.31 A	1.00 A	1.58 A
Leaf group	1	7.40 B	4.39 C	0.54 B	0.91 C
	2	12.2 A	8.11 A	1.81 A	1.30 B
	3	3.69 C	6.06 B	0.23 B	1.83 A
Significance					
DAT		*	***	*	***
Leaf group		***	***	***	***
DAT × leaf group		***	***	***	***

*TPC, yield for total phenolic content (mg GAE kJ^–1^ day); TFC, yield for total flavonoid content (mg CE kJ**^–^**^1^ day); TAC, yield for total anthocyanin content (mg C3GE kJ**^–^**^1^ day); and UAPC, yield for UV-absorbing pigment content (OD kJ**^–^**^1^ day).*

*Leaf groups were determined according to the order of leaf emergence and their relative growth state (Supplementary Figure 3 and Supplementary Table 1).*

*Different letters indicate significant differences by two-way or one-way ANOVA and post-hoc test at P < 0.05 for each parameter referring to the “Materials and Methods” section.*

*P < 0.05; ***P < 0.001.

The UV-B energy yields for the TPC and TFC significantly increased as growth progressed and were higher in leaf group 2 than in the others. As growth progressed, the UV-B energy yields for TPC and TFC decreased in leaf group 3 but increased in leaf group 2. Those for TPC and TFC were the highest in leaf group 2 at 38 DAT. The UV-B yield for the UAPC in both leaf groups 2 and 3 increased significantly as growth progressed and was the highest in leaf group 3 at 38 DAT. The UV-B energy yields for TAC were the highest in leaf group 2 at 38 DAT.

## Discussion

### Narrowband Ultraviolet-B Radiation on Plants

Variables affecting the perception of UV-B radiation by plants, such as dose (fluence rate and duration), timing during a day or growth stages, light sources (solar, broadband, or narrowband UV lamp), and setup (greenhouse or chamber), make it hard to define the intensity or dose thresholds of UV-B radiation (Meyer et al., 2021). This confusion without coherence hampers understanding of plant physiology as well as commercial use of bioactive compound production. High doses of UV-B (high fluence rate, long duration, or shorter wavelength) cause metabolic disorders, such as chlorophyll degradation and the generation of reactive oxygen species (ROS), supporting the long-held opinion that UV-B radiation is harmful to plants (Jordan, 2002). Recent studies have highlighted the regulatory properties of moderate UV-B radiation (low and ecologically relevant level or longer wavelength) as a eustressor and numerous acclimation strategies, including changes in secondary metabolism (Hideg et al., 2013; Neugart and Schreiner, 2018). In general, plants perceive UV-B radiation by the specific receptor UV RESISTANCE LOCUS 8 (UVR8), leading to signal transduction of UVR8-COP1 (CONSTITUTIVE PHOTOMORPHOGENESIS1) with ELONGATED HYPOCOTYL5 (HY5) and HY5 HOMOLOG (HYH) transcription factors (Liang et al., 2019). O’Hara et al. (2019) reported that the expression of a gene encoding the transcription factor ARABIDOPSIS NAC DOMAIN PROTEIN 13 (ANAC13) was induced over a range of UV-B wavelengths at low doses, with a maximum response at 310 nm. Rácz and Hideg (2021) reported that the antioxidant enzyme activities under narrowband 311 nm UV-B radiation evoked opposite responses from broadband UV radiation. The contents of chlorophyll a and chlorophyll b, total chlorophyll, and carotenoid were significantly lower at 23 DAT and were significantly higher in UV6h radiation but lower in UV12h radiation than in the control (Supplementary Figure 4). These suggestions may support the results in this study that the contents of bioactive compounds increased in kale plants exposed to UV-B radiation generated by the narrowband 310 nm UV-B LED for 6 h despite the high fluence rate without distinct damage to growth or chlorophyll content (Figures 4, 5 and Supplementary Figures 1, 4).

### Effect of Ultraviolet-B Radiation on Phenolic Content in Plants

Ultraviolet-B radiation has been reported to be effective in increasing phenolic contents and AOC. In this study, the TPC, TFC, TAC, UAPC, and AOC per DM and per leaf were significantly higher with longer UV-B exposure (Figures 4–6). As a response to UV-B radiation, the enhanced biosynthesis of phenylpropanoids has been well documented in *Brassica* species, including kale (Castillejo et al., 2021; Neugart and Bumke-Vogt, 2021). Under UV-B exposure, phenolics and flavonoids accumulate in epidermal tissues and act as direct UV screens, i.e., the main components of UAP (Neugart and Schreiner, 2018). The innate antioxidant potential of the compounds increases ROS scavenging activity and protects sensitive tissues from the high UV-B energy (Hideg et al., 2013). Dou et al. (2019) reported that AOC was positively correlated with UV-B dose, resulting from linear increases in the TPC and TFC in basil.

Plant and leaf developmental stages determine the UV-B-induced accumulation of bioactive compounds, but the relationships are not straightforward. In this study, leaf groups 1, 2, and 3 correspond to relatively old, intermediate, and young leaves at each growth stage, respectively, as determined by the order of leaf emergence and the relative growth state (Supplementary Figure 3). The increase rates of phenolic accumulation with UV-B radiation were highest in young leaves at 23 DAT, but those were not always higher in younger leaves than the other leaves at 30 and 38 DAT (Figure 6). Rizi et al. (2021) reported that UV-B-exposed young leaves of *Salvia verticillata* showed higher phenylpropanoid production than old leaves as higher gene expression of the key enzymes in their synthesis. However, young leaves are typically located near the top of the plant and are more exposed to UV-B radiation than the other leaves. At the plant canopy levels, higher canopy porosity was positively correlated with the contents of flavonoids such as kaempferol and quercetin in red wine grapes (Martínez-Lüscher et al., 2019). With simulated UVR_*int*_ in individual leaves, the increase rates of TPC and TFC were the highest in intermediate leaves at 30–38 DAT (Figure 6), which is consistent with the previous study (Yoon et al., 2021b). Therefore, at least locally accumulated UV-B-induced phenolics are determined by structural factors such as leaf position, area, and curvature as well as by developmental age.

### Ultraviolet-B Radiation Interception on Plant Structures

The plant structure varies depending on its phyllotaxis and changes as the plant grows, but the plants are exposed to UV-B sources in a one-size-fits-all manner. The UV-B energy absorbed and its physiological interaction with individual leaves inevitably lead to a heterogeneous response in the plant. In this study, UV radiation interception was simulated well along with structural properties, including leaf height, angle, and surface curvature, using ray-tracing simulation and a 3D-scanned plant model (Figure 3). Previous studies on a light interception in plant structures have focused on the spatial distribution of PAR interception and photosynthesis using simulations (Sarlikioti et al., 2011; Shin et al., 2021). In controlled environments, the PAR interception was determined by the physical structures and arrangements of plants and artificial lighting (Kim et al., 2020; Saito et al., 2020). As growth progressed, plant height increased, but the UVR_*int*_ value of leaf group 3 decreased (Figure 3B). Reduced overlap of radiation at the position close to the UV-B LEDs with a narrow radiation area and the mutual shading by neighboring plants decreased the UVR_*int*_ despite growth progress (Yoon et al., 2021b).

### Predicting Models of Phenolic Content in Three-Dimensional Plant Structures

In general, major environmental factors are controlled to optimize crop photosynthesis and growth in controlled environments (Carotti et al., 2021). Secondary metabolite production has been attempted to be maximized beyond biomass controlling environmental factors, including temperature, relative humidity, photoperiod, and light spectrum (Shim et al., 2018; Johnson et al., 2019; Appolloni et al., 2021). Unlike other environmental factors, supplementation with UV-B radiation near harvest could be more effective without growth loss or additional energy input (dos S. Nascimento et al., 2020; Yoon et al., 2020). Since UV-B exposure is a potent abiotic elicitor for the biosynthesis of secondary metabolites, its energy inputs should be optimized (Thakur et al., 2019; Toscano et al., 2019). Optimization of various elicitors has mostly been reported in the studies of *in vitro* plant tissue culture (Ghorbani et al., 2015; Farjaminezhad and Garoosi, 2021).

This study developed statistical models to assess how UV-B radiation as an elicitor enhances the phenolic accumulation in kale plants. For this purpose, the daily absorbed UV-B energy and the phenolic contents were analyzed as each value per leaf (Figures 3C, 6). The developed models suitably explained the TPC, TFC, and UAPC as functions of UV-B radiation interception and leaf order according to growth stage (Table 1 and Figures 7, 8). These models showed high accuracies for predicting the TPC, TFC, TAC, and UAPC (Figure 9). The relatively low *R*^2^ of the models at 23 DAT might be due to the small number of measured samples, which was less than half of the total (Figures 9C,D and Supplementary Figure 2).

Statistical modeling is an effective tool for quantifying the relationship between dependent and independent variables by the statistical significance of their correlations (Kim et al., 2016). Kim et al. (2018) developed a statistical model for glucosinolate content in Chinese cabbage using a second-order multi-polynomial equation with growth duration and temperature as independent variables. In this study, the model structure was determined according to the linear pattern of phenolic accumulation with UV-B dose as shown in the results from previous studies (Dou et al., 2019; Yoon et al., 2021a) or quadratic pattern with leaf group as shown in Figure 6 and the results from a previous study (Yoon et al., 2021b). However, the developed models were limited to kale plants between 23 and 38 DAT in the UVR_*int*_ range below 2.5 W m^–2^ (Figure 3). Within the 3D plant structure, UV-B radiation further enhanced the intraindividual heterogeneity of phenolic contents (Yoon et al., 2021a). Using UV-B radiation interception on a 3D-scanned plant model, the developed models could extend the prediction of a phenolic accumulation from the intraindividual distribution level to the spatial distribution level (Figure 7). This approach to the distribution of metabolites in the 3D plant structure could provide the groundwork for plant metabolism and plant–environment interaction studies (Floros et al., 2017).

### Application to Phenolic Production in the Food System

The main goal of controlled environment agriculture is to maximize plant productivity and minimize practical production costs, including energy costs (Kozai, 2018). The utilization efficiency of lighting systems on plant structures could be calculated using ray-tracing simulations and 3D-scanned plant models (Kim et al., 2020; Saito et al., 2020). The simulation results along with the photosynthesis model could be used to find out the optimal lighting system with the maximal light use efficiency for photosynthesis (Kim et al., 2020). In this study, the UV-B energy yield for phenolic content was calculated (Table 2). The annual production of bioactive compounds was simply estimated based on plant density (plant m^–2^) and cultivation cycle per year according to harvest time (Yoon et al., 2019). From the data in this study, the optimal harvest time for the annual production of TFC was calculated as 30 DAT. Although the plant size and UV-B yield were high at 38 DAT, the annual production was high at 30 DAT because the shorter cultivation period increased the number of cultivations per year. We used kale as the representative plant because the phyllotaxis of the kale is a spiral pattern, which is a common pattern, and is widely cultivated around the world. This model approach could be extended to various plants and structures. Further steps in the modeling procedure for agriculture and food systems were model simulation and model-based analysis (Kim et al., 2016). These steps allow us to investigate numerous scenarios and predict the system responses with cost and input in an effective way (Kreutz and Timmer, 2009). Therefore, the developed model in this study could be used to predict the phenolic accumulation with effective UV-B energy input through model-based simulation in various environments.

## Conclusion

This is the first study on the prediction of phenolic content with UV-B radiation interception and developmental age. Stepwise multiple linear regression models for the TPC, TFC, TAC, and UAPC were developed and validated with high accuracy. Preharvest UV-B radiation was also identified as a suitable strategy for the commercial production of secondary metabolites in controlled environments. Ultimately, the model-based prediction will be used to find out the optimal conditions for the industrial production of secondary metabolites, saving production time and cost.

## Data Availability Statement

The original contributions presented in the study are included in the article/Supplementary Material, further inquiries can be directed to the corresponding author.

## Author Contributions

HY, M-MO, and JS designed the study and prepared the manuscript. HY and JK carried out the data collection. HY performed the model construction, simulation, statistical analysis, and visualization. All authors contributed to the article and approved the submitted version.

## Conflict of Interest

The authors declare that the research was conducted in the absence of any commercial or financial relationships that could be construed as a potential conflict of interest.

## Publisher’s Note

All claims expressed in this article are solely those of the authors and do not necessarily represent those of their affiliated organizations, or those of the publisher, the editors and the reviewers. Any product that may be evaluated in this article, or claim that may be made by its manufacturer, is not guaranteed or endorsed by the publisher.

## Supplementary Material

Supplementary Figure 1 Total fresh mass, leaf area, and root dry mass of kale plants.

Supplementary Figure 2 Leaf fresh mass and leaf area of kale plants.

Supplementary Figure 3 Leaf relative growth rate and expansion rate of leaf populations.

Supplementary Figure 4 Chlorophyll and carotenoid contents in individual leaves of kale plants.

Supplementary Figure 5 Comparison between measured and estimated phenolic contents per leaf of kale plants in the data set.

Supplementary Table 1 Relative growth rate and expansion rate of leaf groups, and the assigned leaf order in kale plants at 23, 30, and 38 DAT.
