# Supplementary material for: Prediction of Phenolic Contents Based on Ultraviolet-B Radiation in Three-Dimensional Structure of Kale Leaves
Source: Front Plant Sci. 2022 Jun 9;13:918170. doi: 10.3389/fpls.2022.918170 (PMC9228028; doi:10.3389/fpls.2022.918170)
Supplement: Supplementary Figure 1 — Total fresh mass, leaf area, and root dry mass of kale plants. [file Data_Sheet_1.docx]

Supplementary Materials

# Total growth parameter

Total fresh mass, leaf area, and root dry mass were measured separately for kale plants harvested at 15, 23, 30, and 38 DAT with four plants per treatment (**Supplementary Figure 1**). All parameters were not significantly different among the treatments by one-way ANOVA at *P* < 0.05.


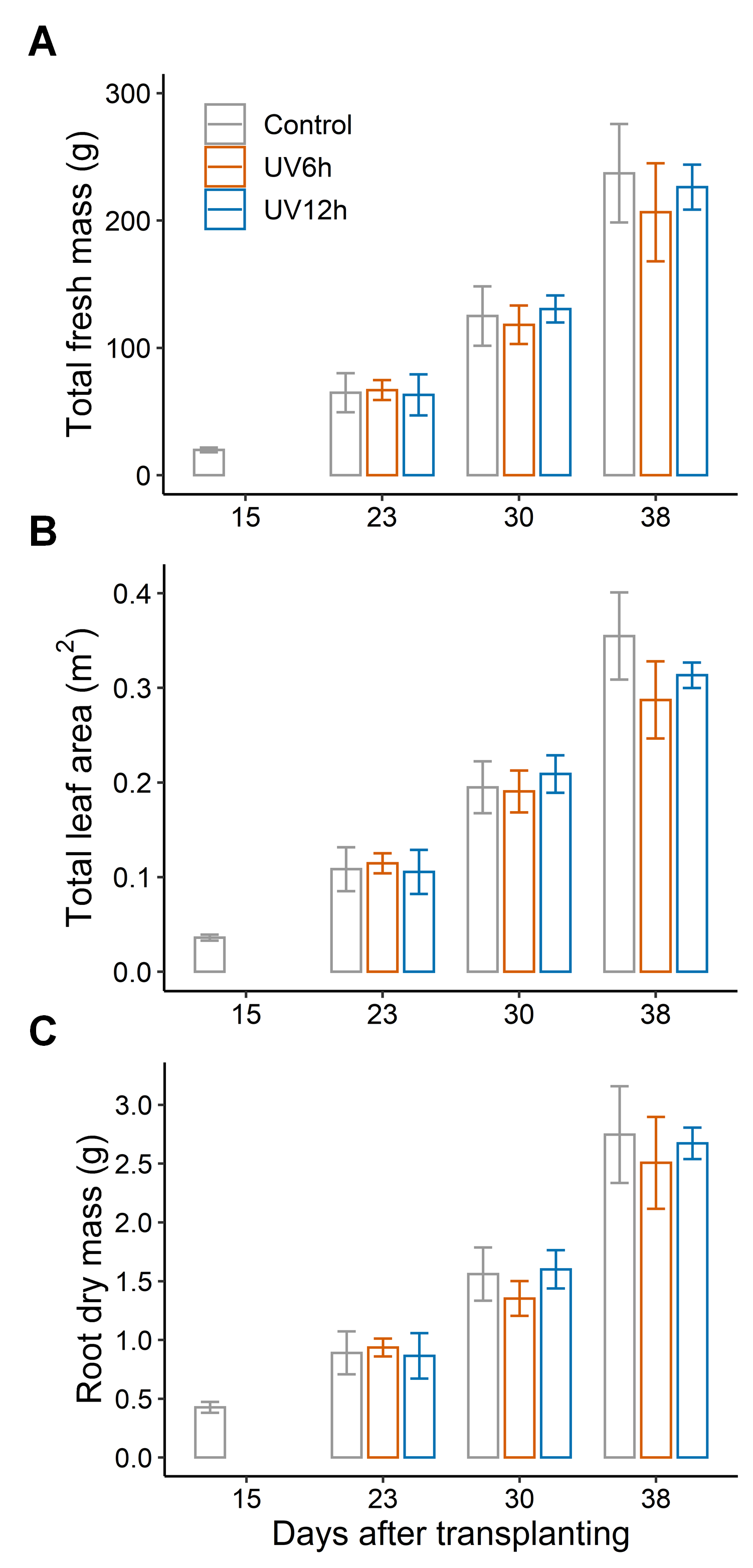


**Supplementary Figure 1.** Total fresh mass **(A)**, leaf area **(B)**, and root dry mass **(C)** of kale plants according to UV-B radiation and growth stage at 15, 23, 30, and 38 days after transplanting. Vertical bars indicate SD, *n* = 4.

# Determination of leaf group according to growth stage based on relative growth rate

Leaf groups at each growth stage were characterized with the changes of leaf fresh mass and leaf area over a time span of 7–8 days. Leaf fresh mass and leaf area were measured separately for each leaf in kale harvested at 15, 23, 30, and 38 DAT with four plants per treatment (**Supplementary Figure 2**). The relative growth rate (RGR) and relative expansion rate (RER) were calculated as (Behn et al., 2011; Pontarin et al., 2020):

RGR (g g^–1^ d^–1^) = [ln(FM_t2_) – ln(FM_t1_)]/(t_2_ – t_1_) Eq. S1

RER (cm cm^–1^ d^–1^) = [ln(LA_t2_) – ln(LA_t1_)]/(t_2_ – t_1_) Eq. S2

where FM is the individual leaf fresh mass (g), LA is the individual leaf area (cm^2^) at DAT = t, and t is the time span (day). In this study, leaf RGR and RER were calculated between sampling days at 15–23, 23–30, and 30–38 DAT. When the leaf did not appear at the prior time, their RGR and RER values were inferred by interpolation by fitting a regression. The values were regressed with quadratic function of leaf order (*L*), and all R^2^ was 0.99 (**Supplementary Figure 3**).

RGR (23 DAT) = 0.119 + 0.318*L* + 0.100*L^2^*

RGR (30 DAT) = 0.076 + 0.244*L* + 0.088*L^2^*

RGR (38 DAT) = 0.052 + 0.249*L* + 0.110*L^2^*

RER (23 DAT) = 0.103 + 0.280*L* + 0.094*L^2^*

RER (30 DAT) = 0.062 + 0.208*L* + 0.081*L^2^*

RER (38 DAT) = 0.040 + 0.202*L* + 0.102*L^2^*

On all these dates, the older leaves presented RGR and RER values close to zero, and their values did not change between the dates. As the growth progressed, the gaps in both RGR and RER values between the leaves narrowed. Based on these patterns, the leaf populations at each growth stage were divided into three leaf groups. The RGR and RER values of leaf groups and the assigned leaf order at each growth stages are shown in **Supplementary Table 1**.


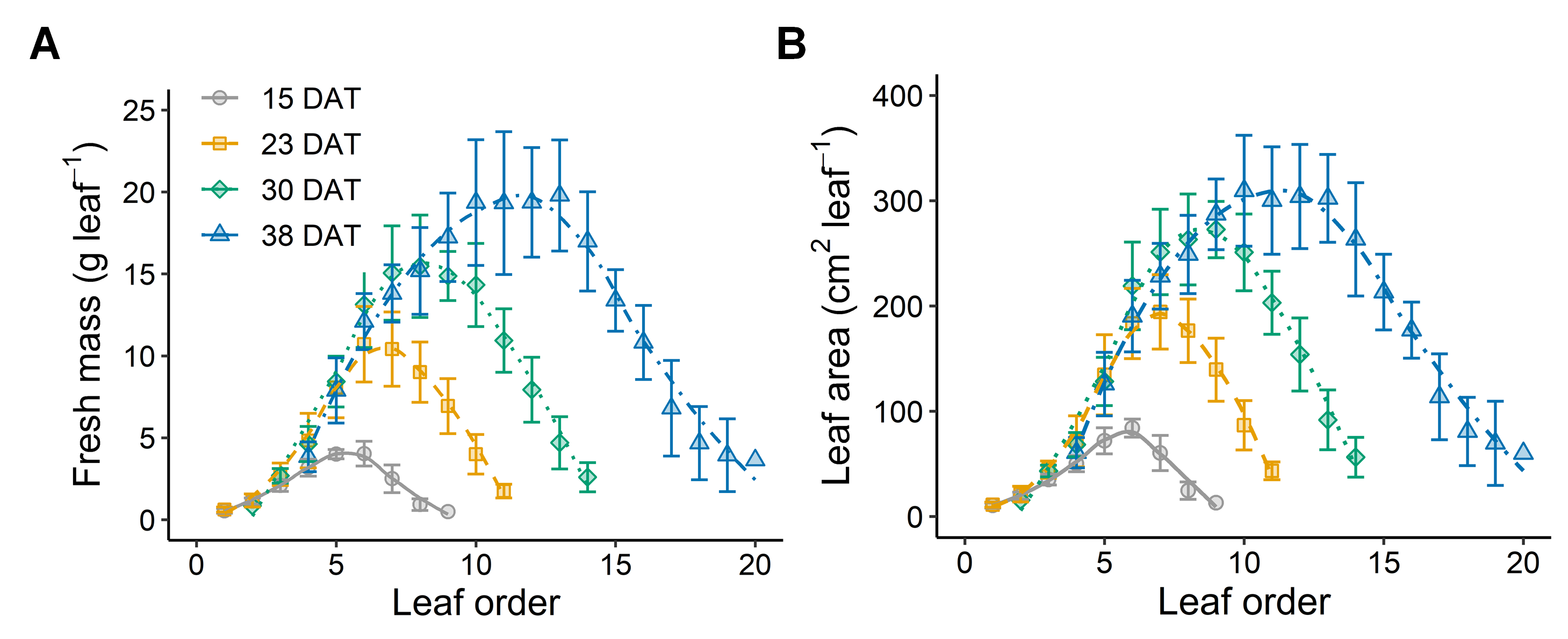


**Supplementary Figure 2.** Leaf fresh mass **(A)** and leaf area **(B)** of kale plants according to leaf order and growth stage at 23, 30, and 38 days after transplanting (DAT).


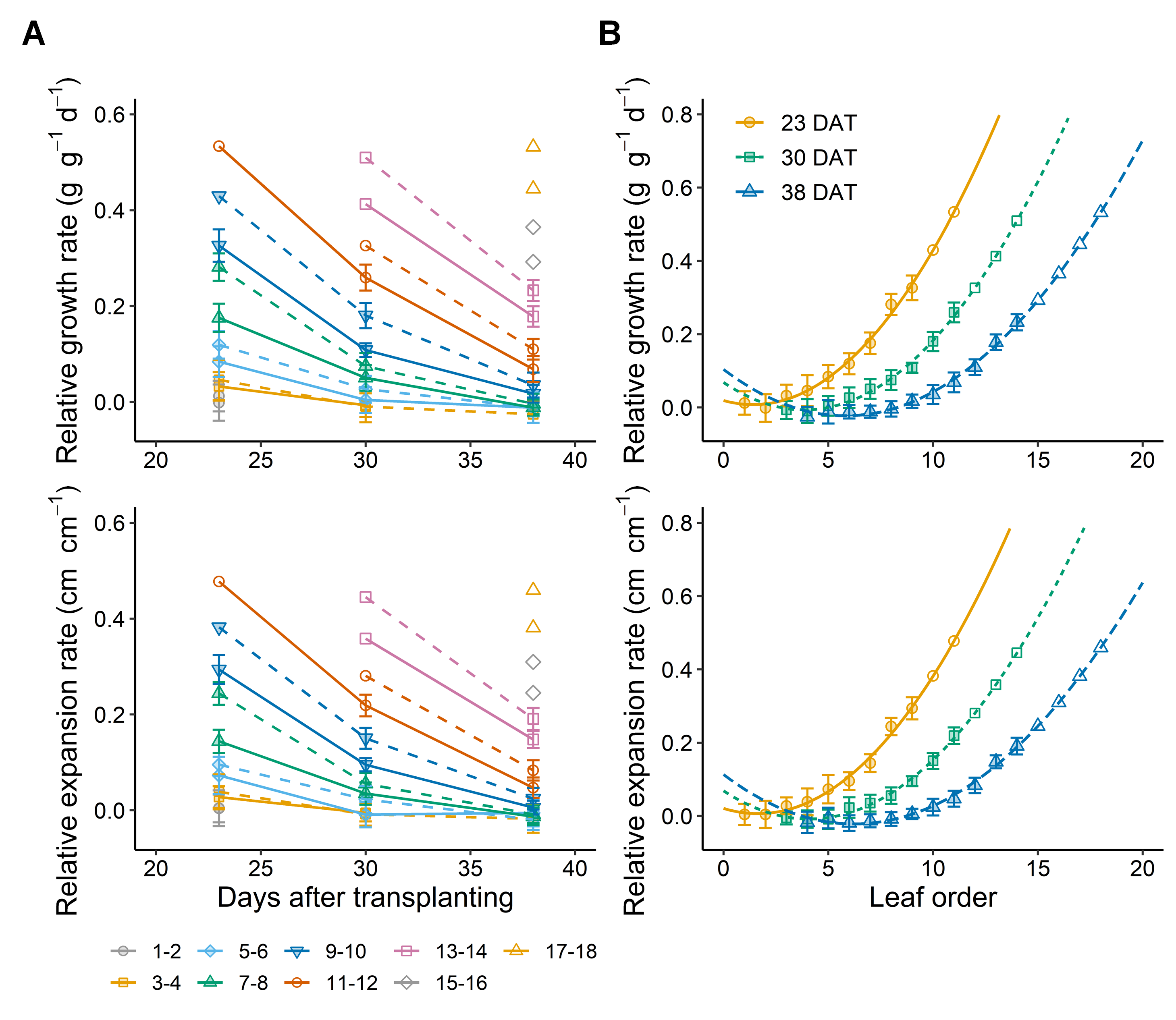


**Supplementary Figure 3.** Leaf relative growth rate and expansion rate of leaf populations in kale plants according to 23, 30, and 38 days after transplanting (DAT) **(A)** and leaf order **(B)**. Open points indicate interpolated values by fitting a regression since the leaf did not appear at the prior time. All R^2^ of regression lines with leaf order in the left panels were 0.99. The solid and dash lines in the right panels indicate odd and even leaf orders (the absolute order of leaf emergence), respectively.

**Supplementary Table 1.** Relative growth rate (RGR) and relative expansion rate (RER) of leaf groups, and the assigned leaf order in kale plants at 23, 30, and 38 days after transplanting (DAT).

| DAT | Leaf group | RGR (g g^–1^ d^–1^) | RER (cm cm^–1^ d^–1^) | Leaf order |
| --- | --- | --- | --- | --- |
| 23 | 1 | < 0.04 | < 0.3 | 1-3 |
|  | 2 | 0.05-0.18 | 0.04-0.15 | 4-7 |
|  | 3 | 0.28-0.53 | 0.24-0.48 | 8-11 |
| 30 | 1 | < 0.03 | < 0.03 | 3-6 |
|  | 2 | 0.05-0.11 | 0.04-0.10 | 7-9 |
|  | 3 | 0.18-0.51 | 0.15-0.45 | 10-14 |
| 38 | 1 | < 0.01 | < 0.01 | 4-8 |
|  | 2 | 0.02-0.11 | 0.01-0.08 | 9-12 |
|  | 3 | 0.18-0.53 | 0.15-0.46 | 13-18 |

RGR and RER values were obtained at 15–23, 23–30, and 30–38 DAT, and referred to Eqs. S1, S2, and Supplementary Figures 1, 2.

# Chlorophyll and carotenoid contents according to UV-B radiation and growth stage


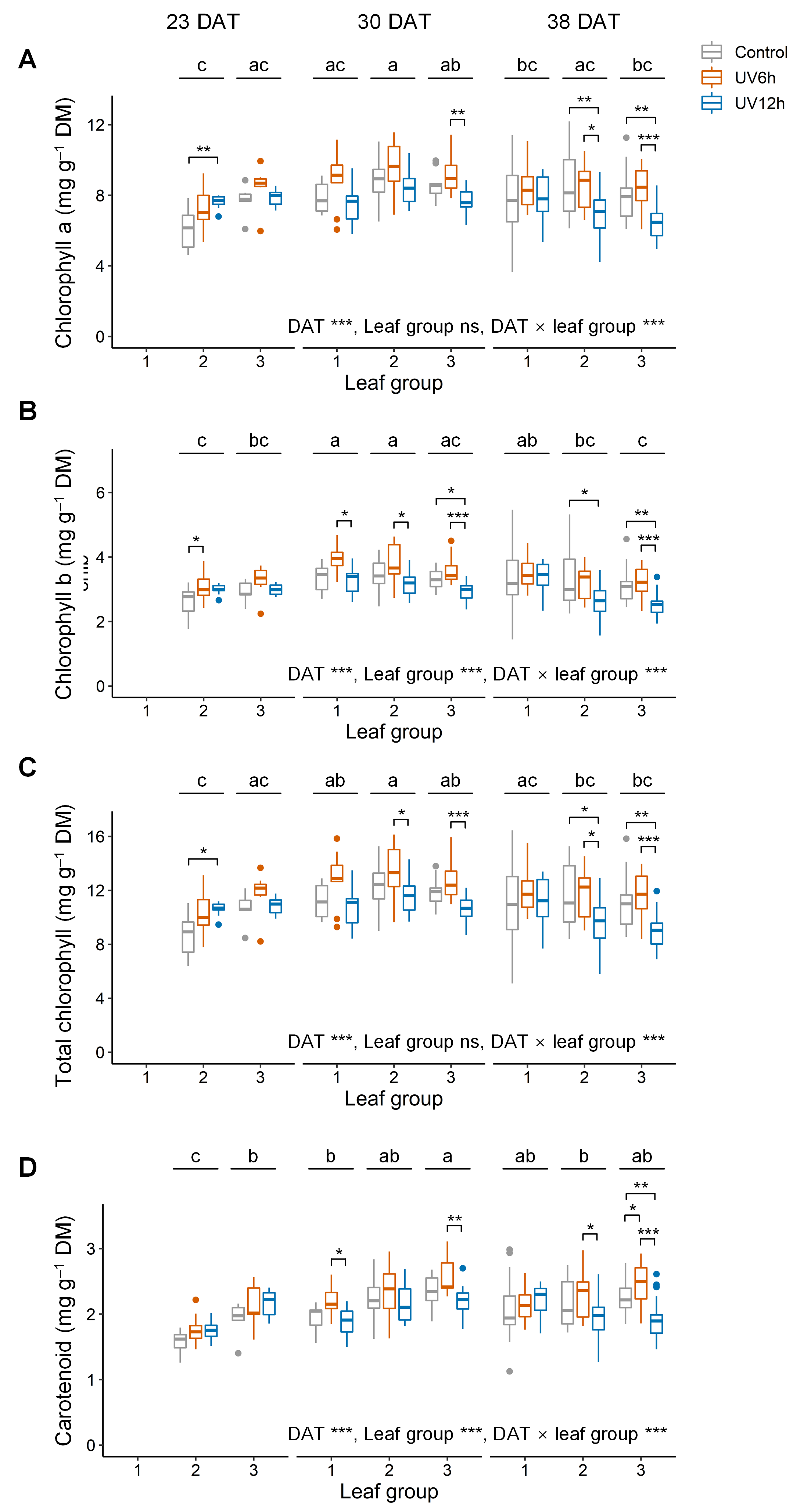


**Supplementary Figure 4.** Chlorophyll a (**A**) and b (**B**), total chlorophyll (**C**), and carotenoid (**D**) contents in individual leaves of kale plants according to UV-B radiation and leaf group at 23, 30, and 38 days after transplanting (DAT). Asterisks indicates significant differences between UV-B treatments at each growth stage and leaf group by one-way ANOVA and Tukey’s HSD test, *, *P* < 0.05; **, *P* < 0.01; ***, *P* < 0.001; *n* = 6–22. Different letters indicate significant differences among growth stage and leaf group at *P* < 0.05 by two-way ANOVA and post-hoc test (*n* = 19–58) referring to Materials and Methods.

# Comparisons of measured and estimated phenolic contents per leaf

Multiple regression models for predicting the phenolic contents were obtained by stepwise regression using backward elimination method based on a second-order multi-polynomial model in Eq. 1. The regression models were selected with significance of all independent variables, and finally developed models are shown in Figure 4 and Table 1. In the data set using the model development, the measured and estimated contents were compared across all growth stages (**Supplementary Figure 5**). The R^2^ for the four models was higher in the data set integrated from the models across whole growth stage than in the models at each growth stage (Table 1).


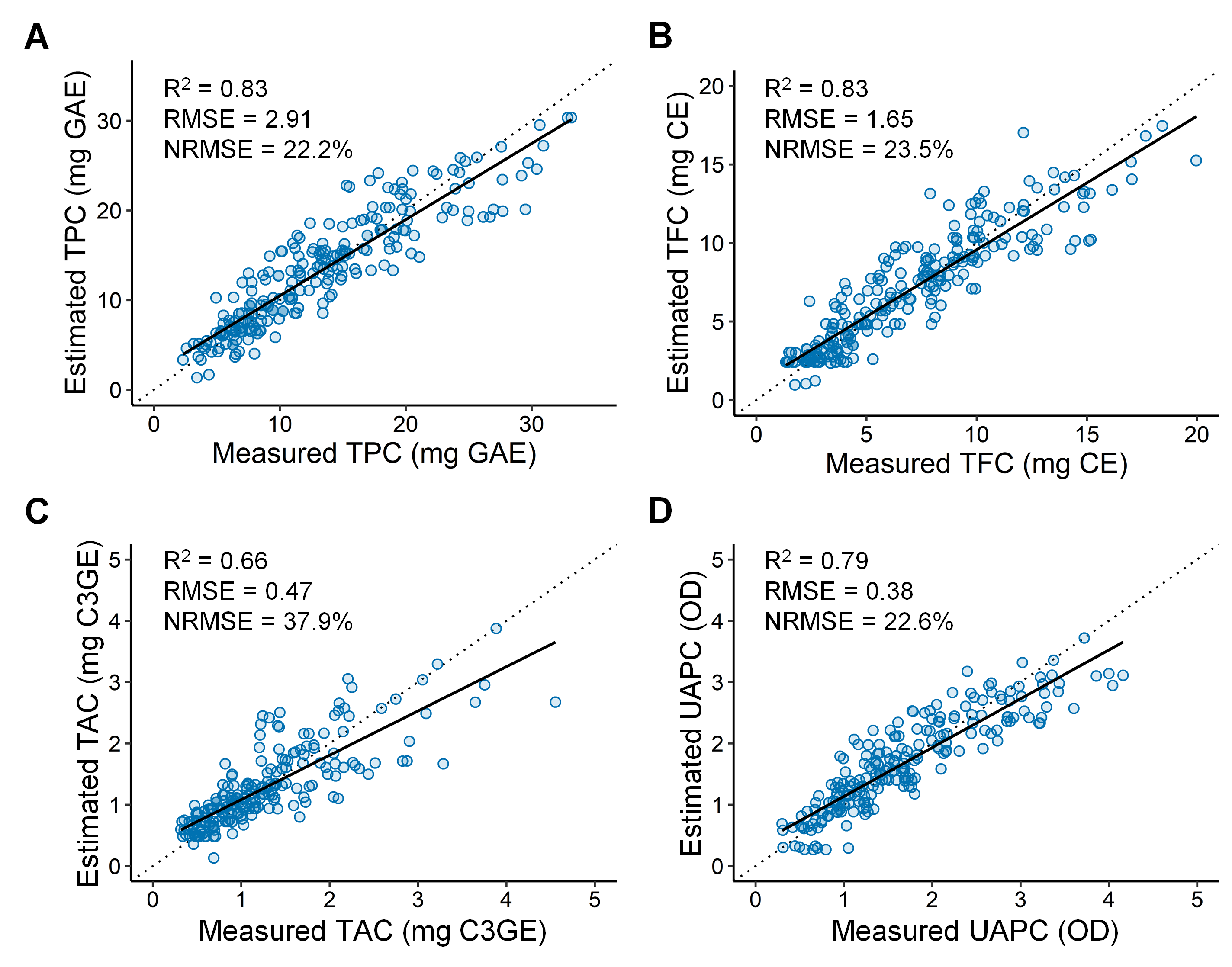


**Supplementary Figure 5.** Comparison between measured and estimated phenolic contents per leaf of kale plants in the data set integrated from the models across all growth stages. TPC, total phenolic content **(A)**; TFC, total flavonoid content **(B)**; TAC, total anthocyanin content **(C)**; UAPC, UV-absorbing pigment content **(D)**. The coefficient of determination (R^2^), root mean squared error (RMSE), and the normalized RMSE (NRMSE) are presented inside each panel.
